# Women with breast cancer taking chemotherapy: depression symptoms and treatment adherence

**DOI:** 10.1590/0104-1169.3564.2491

**Published:** 2014

**Authors:** Bianca Fresche de Souza, Jéssica Andrade de Moraes, Aline Inocenti, Manoel Antônio dos Santos, Ana Elisa Bauer de Camargo Silva, Adriana Inocenti Miasso

**Affiliations:** 1 Undegraduate student in Pharmacy Biochemistry, Faculdade de Ciências Farmacêuticas de Ribeirão Preto, Universidade de São Paulo, Ribeirão Preto, SP, Brazil. Scholarship holder of the Scientific Initiation Program at the Conselho Nacional de Desenvolvimento Científico e Tecnológico (CNPq), Brazil; 2 MSc, RN, Prefeitura Municipal da Estância Turística de Batatais, Batatais, SP, Brazil; 3 PhD, Associate Professor, Faculdade de Filosofia, Ciências e Letras de Ribeirão Preto, Universidade de São Paulo, Ribeirão Preto, SP, Brazil; 4 PhD, Professor, Faculdade de Enfermagem, Universidade Federal de Goiás, Goiânia, GO, Brazil; PhD, Professor, Departamento de Enfermagem Psiquiátrica e Ciências Humanas, Escola de Enfermagem de Ribeirão Preto, Universidade de São Paulo, WHO Collaborating Centre for Nursing Research Development, Ribeirão Preto, SP, Brazil

**Keywords:** Breast Neoplasms, Drug Therapy, Depression, Medication Adherence

## Abstract

**OBJECTIVE:**

to verify depressive symptoms and adherence to chemotherapy among women with breast cancer who are served by the Pharmacy of the Chemotherapy Center of a university hospital.

**METHOD:**

cross-sectional study with quantitative approach conducted with 112 women receiving chemotherapy. Structured interviews guided by a script addressing socio-demographic, clinical and therapeutic information, the Morisky Test, and the Beck Depression Inventory were used to collect data.

**RESULTS:**

12.50% and 1.78% of the patients experienced "moderate" and "severe" depression, respectively, while 10.59% did not use antidepressant medication. A statistically significant association was found between levels of depression and the use of antidepressants. Lack of adherence was identified in 46.43% of the participants.

**CONCLUSION:**

these findings show the need to regularly screen for depressive symptoms and for adherence to chemotherapy treatment among women with breast cancer, in order to provide early detection and appropriate treatment centered on patients, and to improve their quality of life.

## Introduction

Breast cancer is the malignant neoplasia accounting for the highest number of deaths among women in Brazil and in the world. A total of 52,680 new cases were estimated in Brazil for 2012, i.e., an incidence of 52.5/100,000 women^(^ 1 ^)^.

Physical and psychological changes are evident and impactful from the time the diagnosis is revealed and, even though efficient treatments are available and survival rates have significantly improved in recent years, cancer is still seen as a distressful disease with a potential risk of death. Patients are faced with the imminent loss of a body part highly invested in feminine representations and the fear of an incurable disease, which results in suffering and stigmatization^(^ 2 ^)^.

Many factors are associated with the triggering of psychosocial and physical stress in patients with breast cancer: surgery, coadjunvant treatments, fear of recurrence and death, bodily changes, reduced femininity and sexuality^(^ 2 ^)^. These changes are frequently accompanied by depression, a psychiatric morbidity that is common during and after treatment of this type of cancer^(^ 3 ^)^.

In this regard, the literature shows that depressive symptoms are recurrent in patients with clinical diseases, including those with breast cancer. These symptoms, experienced by about 22% of patients^(^ 4 ^)^, may progress to a chronic condition and prevent patients from performing daily tasks. In more severe cases, this condition may lead to suicide. One study reports that pain and depression were associated with the risk of suicide among patients with cancer^(^ 5 ^)^. Monitoring patients with the purpose of identifying behavioral traits, including suicidal thoughts, is essential to grounding both pharmacological and non-pharmacological approaches^(^ 6 ^)^. 

Even though depression is recurrent among cancer patients, it often goes unnoticed, or when a diagnosis is reached, the condition is not properly treated. Only 35% of the patients are properly diagnosed and treated^(^ 7 ^)^. This is a relevant factor, because depression is associated with a worse prognosis and higher mortality due to cancer^(^ 8 ^)^. 

Cancer patients with depressive symptoms also tend to present less adherence to treatments, and consequently with a worse prognosis^(^ 5 ^)^. In addition to depressive symptoms, the literature shows that various factors account for low adherence among cancer patients, including adverse effects from medication, the need for significant behavioral change, poor communication between patients and health workers, inefficiency in health services, the complexity of therapeutic schemes, patient dissatisfaction with care delivery, insufficient social support, and patient beliefs concerning treatment, among other factors^(^ 9 ^)^. Non-adherence to treatment among breast cancer patients is associated with worse clinical outcomes, indicating the importance of screening.

All the aforementioned aspects point to the importance of investigating depressive symptoms and adherence to chemotherapy among breast cancer patients to optimize treatment and improve patients' quality of life.

## Objective

To verify depressive symptoms and adherence to chemotherapy among women with breast cancer served by the Pharmacy of the Chemotherapy Center of a large university hospital.

## Method

This analytical, cross-sectional study with a quantitative approach was conducted in the Pharmacy of the Chemotherapy Center of a large university hospital located in the interior of São Paulo, Brazil. This pharmacy provides free-of-cost oral and injectable chemotherapy medication to patients in follow-up in the facility.

The convenience sample was composed of 112 patients with a breast cancer diagnosis who received their chemotherapy at the pharmacy of the Chemotherapy Center from October 2012 to March 2013. Inclusion criteria were: having a medical diagnosis of breast cancer; having a prescription for chemotherapy medication; and being 18 years old or older. All the patients who attended the service in the period of data collection and met the inclusion criteria were invited to participate in the study.

Structured interviewing was the technique used to collect data. Interviews were held in a private room in the facility and were based on a script composed of three parts. The first addressed the patients' demographic, socioeconomic, clinical and therapeutic variables. In regard to the medication, their description was based on the first level of the Anatomical Therapeutic Chemical (ATC) classification system of the WHO (www.whocc.no/atc_ddd_index).

The second part of the script was composed of the Morisky Test^(^ 10 ^)^, which enables identifying patient adherence to medication therapy and assesses behavior concerning the daily use of medication. The instrument is composed of four questions:* Do you sometimes forget to take your medicine?; Are you careless at times about taking your medicine?; When you feel better, do you sometimes stop taking your medicine?; and If you feel worse when you take medicine, do you sometimes stop taking it?*

Adherence was measured using dichotomous answers: "No" and "Yes", to which 0 (zero) and 1 were respectively assigned. Hence, 1 was assigned to positive answers and 0 to negative answers. With the purpose to compare and discuss results, we established criteria to classify levels of adherence: more adherent for patients who obtained a score of 0 on the Morisky test, and less adherent for those who scored from 1 to 4 on the instrument.

The script's third part was intended to identify the presence of depressive symptoms in the study sample. For that, the Beck Depression Inventory was used^(^ 11 ^)^. This instrument consists of a self-report scale for symptoms, composed of 21 items with different alternatives for answers concerning how the individual has felt in the last week, including the date upon which the instrument was applied. These items correspond to different levels of severity of depressive symptoms. The sum of the items' scores results in an overall score that permits establishing the intensity of depressive symptoms: "no depression", "dysphoria", "moderate depression", and "severe depression". The choice of the cut off point appropriate for each level depends on the nature of the sample and the study's objectives. For samples not diagnosed with depression, as is the case in this study, scores above 15 are recommended to detect "dysphoria" and the term "depression" is designated for individuals with scores above 20.

After the instruments were applied, data related to adherence and to the Beck Depression Inventory were analyzed using the EpiInfo(tm) program, version 3.2, in the public domain (http://www.cdc.gov/epiinfo). Potential associations between dependent and independent variables were verified through Fisher's exact test whenever the frequency of the patients was below five. The Chi-square test was used whenever the frequency was above five. For that, the variables were then dichotomized, adopting a level of significance of α= 0.05.

The project was approved by the Institutional Review Board at the institution where the study was developed (Process HCRP No. 6349/2010) and the participants signed free and informed consent forms in accordance with Resolution 196/96, Brazilian Council of Health.

## Results

A total of 112 women aged between 26 and 90 years old participated in the study. There were a greater percentage of married (47.32%) and retired (34.82%) women, and homemakers (28.57%).

Most patients undergoing treatment used only one chemotherapy medication (94.64%), medication classified as L02B- hormone antagonists and related agents (98.21%), and had undergone only one surgery in their lifetime (58.04%) (Table 1).

**Table 1:** - Distribution of the study participants according to clinical and therapeutic variables. Ribeirão Preto, SP, Brazil, 2013

Variables		n	%
Number of medications			
	One medication	106	94.64
	More than one medication	6	5.36
ATC Classification			
	Alkylating agents (L01A)	2	1.78
	Antimetabolites (L01B)	1	0.89
	Plant alkaloids and other natural products (L01C)	2	1.78
	Cytotoxic antibiotics and related substances (L01D)	2	1.78
	Other anticancer agents (L01X)	1	0.89
	Hormones antagonists and related agents (L02B)	110	98.21
Number of surgeries			
	Up to one surgery	65	58.04
	More than one surgery	47	41.96
Time since diagnosis			
	1 – 30 months	56	50.00
	31 – 144 months	56	50.00
	Total	112	100.00

**Figure 1:**
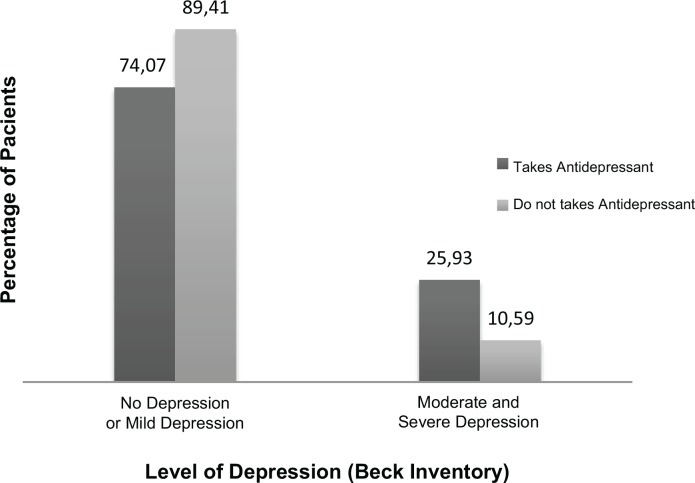
- Distribution of the study's participants according to the intensity of depressive symptoms and use of anti-depressive medication

The results indicate that 12.50% and 1.78% of the patients, respectively, presented "moderate" or "severe" depression according to the classification of intensity of depressive symptoms recommended by the Beck Depression Inventory^(^ 11 ^)^. Because anti-depressive medication is usually prescribed to patients with moderate or severe depression, we opted to dichotomize the intensity of depressive symptoms into "no depression or with dysphoria" and "moderate or severe depression" to present data concerning the use of this therapeutic classification. Note that 10.59% of the patients classified with "moderate" or "severe" depression did not take anti-depressive medication and 25.93% presented depressive symptoms even though they were taking antidepressants. Statistically significant association was found between the intensity of depressive symptoms and the use of anti-depressants (χ^2^=0.998; p=0.04). Note that 74.07% of the patients using anti-depressants presented no depressive symptom or presented only mild symptoms (Figure 1).

Data analysis revealed no statistically significant association among intensity of depressive symptoms and the demographic, socioeconomic, clinical or the therapeutic variables investigated in this study. It is worth noting, however, that a higher percentage of moderate to severe depression was found among women aged up to 36 years old (19.64%), with income (16.89%), with more than three dependents (22.22%), and among those who undergone two or more surgeries (17.39%) (Table 2).

**Table 2:** - Prevalence of depressive symptoms according to demographic, socioeconomic, clinical and therapeutic variables. Ribeirão Preto, SP, Brazil, 2013

Variables		Dysphoria and no depression	Moderate and severe depression	Total	p
		N (%)	N (%)	N(%)	
Age					0.10
	Up to 56 years old	45 (80.36)	11 (19.64)	56 (100.00)	
	57 years old or older	51 (91.07)	05 (8.93)	56 (100.00)	
Marital status					0.64
	Partner	48 (87.27)	07 (12.73)	55 (100.00)	
	No partner	48 (84.21)	09 (15.79)	57 (100.00)	
Schooling					0.65
	Completed middle school	87 (86.14)	14 (13.86)	101 (100.00)	
	Bachelor’s or graduate studies	09 (81.81)	02 (18.19)	11 (100.00)	
Occupation					0.38
	With income	64 (83.11)	13 (16.89)	77 (100.00)	
	No income	32 (91.43)	03 (8.57)	35 (100.00)	
*Per capita* income					0.87
	Up to 400 Brazilian Reais	52 (85.25)	09 (14.75)	61 (100.00)	
	More than 400 Brazilian Reais	44 (86.27)	07 (13.73)	51 (100.00)	
Number of dependents					0.17
	Up to 3 dependents	75 (88.23)	10 (11.77)	85 (100.00)	
	More than 3 dependents	21 (77.78)	06 (22.22)	27 (100.00)	
Number of surgeries					0.43
	Up to one surgery	58 (87.88)	08 (12.12)	66 (100.00)	
	Two or more surgeries	38 (82.61)	08 (17.39)	46 (100.00)	
Number of medications					1.00
	Up to one medication	91 (85.85)	15 (14.15)	106 (100.00)	
	More than one medication	05 (83.33)	01(16.67)	06 (100.00)	
Time since diagnosis					0.80
	Up to 12 months	17 (85.00)	03 (15.00)	20 (100.00)	
	From 13 to 36 months	45 (83.33)	09 (16.67)	54 (100.00)	
	From 37 to 60 months	18 (85.71)	03 (14.29)	21 (100.00)	
	More than 60 months	16 (94.12)	01 (5.88)	17 (100.00)	

Number of surgeries 0.43

**Figure 2:**
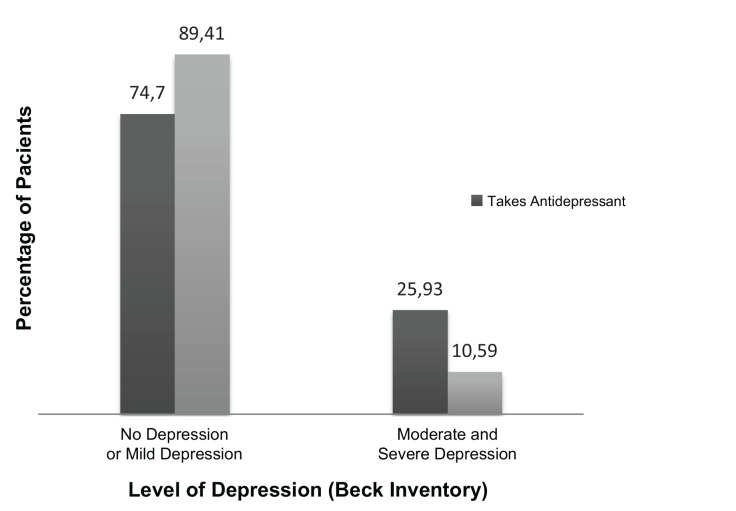
- Distribution of the study's participants according to the classification of symptoms and adherence to chemotherapy treatment

A total of 46.43% of the patients failed to adhere to the medication prescribed to treat their cancer. Note that 5.35% presented "moderate" or "severe" depression. Most of the patients without depressive symptoms were adherent to medication (Figure 2).

No significant association was found between adherence to chemotherapy and the studied variables (p>0.05), though a higher percentage of adherence was found among women aged 57 years old or older (57.14%), with no partner (56.14%), with income (57.14%), and among those whose diagnosis was up to 12 months prior (70.00%) (Table 3).

**Table 3:** - Prevalence of adherence to chemotherapy therapy according to demographic, socioeconomic, clinical and therapeutic variables. Ribeirão Preto, SP, Brazil, 2013

Variable		Adherent	No Adherent	Total	p
		N (%)	N (%)	N (%)	
Age					
	Up to 56 years old	27 (48.21)	29 (51.79)	56 (100.00)	0.34
	57 years old or older	32 (57.14)	24 (42.86)	56 (100.00)	
Marital status					
	Partner	27 (49.09)	28 (50.91)	55 (100.00)	0.45
	No partner	32 (56.14)	25 (43.86)	57 (100.00)	
Schooling					
	Completed middle school	52 (51.49)	49 (48.51)	101 (100.00)	0.53
	Bachelor’s or graduate studies	07 (63.64)	04 (36.36)	11 (100.00)	
Occupation					
	With income	44 (57.14)	33 (42.86)	77 (100.00)	0.16
	No income	15 (42.86)	20 (57.14)	35 (100.00)	
*Per capita *income					
	Up to 400 Brazilian Reais	31 (50.82)	30 (49.18)	61 (100.00)	0.66
	More than 400 Brazilian Reais	28 (54.90)	23 (45.10)	51 (100.00)	
Number of dependents					
	Up to 3 dependents	44 (51.76)	41 (48.24)	85 (100.00)	0.73
	More than 3 dependents	15 (55.56)	12 (44.44)	27 (100.00)	
Number of surgeries					
	Up to 1 surgery	34 (51.51)	32 (48.49)	66 (100.00)	0.76
	More than 2 surgeries	25 (54.35)	21 (45.65)	46 (100.00)	
Number of medications					
	Up to 1 medication	56 (52.83)	50 (47.17)	106 (100.00)	1.00
	More than 1 medication	03 (50.00)	03 (50.00)	06 (100.00)	
Time since diagnosis					0.12
	Up to 12 months	14 (70.00)	06 (30.00)	20 (100.00)	
	From 13 to 36 months	26 (50.00)	26 (50.00)	52 (100.00)	
	From 37 to 60 months	10 (45.46)	12 (54.54)	22 (100.00)	
	More than 60 months	09 (50.00)	09 (50.00)	18 (100.00)	

## Discussion

This study investigated depressive symptoms and adherence to chemotherapy treatment among women with breast cancer. We verified that 12.50% and 1.78% of the patients presented "moderate" or "severe" depression, respectively, according to the classification adopted by Beck^(^ 11 ^)^. The literature shows that women with breast cancer undergoing chemotherapy present a higher rate of depression^(^ 12 ^)^. The factors contributing to depression include sleep disorders, menopausal symptoms, nausea, and pain caused by the high levels of proinflammatory cytokines, due to the tissue damage that results from chemotherapy and radiotherapy^(^ 13 ^)^. It is important to keep in mind that the rapid decrease of estrogen during chemotherapy also favors depressive symptomatology, because this hormone increases the brain's sensitivity to serotonin^(^ 14 ^)^.

Depression is a chronic condition associated with high levels of functional impairment, negative development of concomitant clinical diseases, severe psychological and physical distress, which may result in suicide^(^ 5 ^)^. An alarming fact in this context is that 10.59% of the patients who did not use anti-depressant medication presented "moderate" to "severe" depression.

Note that even taking medication, 25.93% of the patients still presented depressive symptoms. A possible explanation for this finding is the time antidepressant medication has been in use, that is, less than the time necessary to obtain therapeutic results, a variable that was not investigated in this study. The literature shows, however, that about 30% to 50% of depressive conditions do not satisfactorily respond to the first treatment even when it is properly implemented, regardless of the medication prescribed^(^ 15 ^)^. A study that investigated some depressive symptoms in a sample of patients with a diagnosis of depression and in continuous use of medication reports that 42% of the individuals still experience severe symptoms^ (16)^.

The described aspects reveal the importance of using psychosocial interventions, in addition to medication therapy, when treating depression for patients to achieve complete recovery and improved quality of life^(^ 17 ^)^. In this case, psychotherapy, with its different approaches, can be used either as the primary treatment or as an adjuvant to pharmacotherapy, especially when considering the characteristics of the treatment delivered to cancer patients.

Statistical significance between intensity of depressive symptoms and the use of antidepressant medication was found. Most patients (74.07%) using antidepressants did not experience depressive symptoms or only experienced mild symptoms. The importance of antidepressants for this population is also related to its efficacy in treating other symptoms frequently associated with breast cancer, including fatigue, difficulties sleeping and hot flashes^(^ 18 ^)^. 

In regard to the relationship between depressive symptoms and the variables investigated in this study, we note that the highest percentages of "moderate" and "severe" depression were found among women up to the age of 56 years, but there is no consensus in the literature of how age is related to depression. One study, however, shows intense concern on the part of young women with breast cancer in regard to chemotherapy because it reduces the production of female hormones, which may result in early menopause with a consequent infertility, in addition to the difficulty dealing with the psychological stress related to the partial or total removal of breasts^(^ 6 ^)^.

This study reveals that 46.3% of the patients did not adhere to the chemotherapy medication prescribed to treat their cancer. This percentage is high considering the consequences of such a behavior, because lower adherence levels are associated with increased risk of death^(^ 19 ^)^.

One study reveals that non-adherence to tamoxifen was associated with lower rates of survival, free of the disease^(^ 20 ^)^. This is relevant information for this study because most of the investigated patients (98.21%) used medications classified as L02B- hormone antagonist and related agents, and tamoxifen was the medication most frequently prescribed. Tamoxifen is an estrogen hormone adjuvant used for more than 25 years to treat women with breast cancer with proven efficiency for the reduction of mortality and in preventing recurrences. The use of tamoxifen, compared to no treatment, reduces the risk of recurrence for about 15 years^(^ 21 ^)^.

The factors usually indicated as those contributing to non-adherence in breast cancer patients include the therapy's adverse effects. Hormone therapy, including tamoxifen, may cause hot flashes, fluid retention, bleeding, skin rashes, vaginal itching and dryness, the risk of endometrial cancer, joint pain, or deep vein thrombosis, among other^(^ 22 ^)^.

No statistically significant association was found between adherence to chemotherapy and the studied variables. Note, however, that higher levels of adherence were found among women 57 years or older and those with no partners. In regard to age, one study corroborates this study's results, as it reports higher adherence among older women^(^ 9 ^)^. Nonetheless, there is no consensus in the literature in regard to this aspect. The higher level of adherence among patients without a partner conflicts with results in the literature, which suggests that the presence of a partner is associated with higher levels of adherence^(^ 23 ^)^, thus, further research is needed to investigate the relationship between these variables.

 A total of 5.35% of the patients who did not adhere to medication experienced moderate or severe depression. There is evidence that depression may be associated with non-adherence to treatment^(^ 7 ^)^. Even though antidepressant medication is required for these patients, the medication to be prescribed to patients has to be carefully chosen. Selective serotonin reuptake inhibitors (SSRIs) are widely used; they may, however, inhibit cytochrome P450 2D6, necessary to activate tamoxifen, interfering in the efficiency of this medication for the prevention of breast cancer recurrence. It is currently believed that Citalopram, and possibly other SSRIs with lower potency, may be prescribed to these patients without negatively affecting the result of the adjuvant therapy with tamoxifen^(^ 24 ^)^. The possibility of such a pharmacological intervention suggests that oncologists and psychiatrics need to work together.

It is important to note that this study's results should be considered while bearing in mind its methodological limitations: the use of a convenience sample and the fact the study was conducted in a single facility with outpatients, which limits the generalization of results to other groups. 

## Conclusion

This study investigated depressive symptoms and adherence to chemotherapy treatment among women with breast cancer. A high percentage of non-adherent patients with intense depressive symptoms such as "moderate" and "severe" depression was found.

The identification of women with depressive symptoms, despite the use of antidepressants, is an important finding given the influence of such symptoms, both on treatment adherence and on the progression of cancer. This result indicates that other therapeutic modalities are required in addition to a psychopharmacological approach.

These results also show the importance of evaluating the antidepressant medication most appropriate to the patient while taking into account the anticancer treatment she will undergo, because the class of antidepressants may be related both to the improvement of symptoms attributed to the disease and to the chemotherapy treatment, such as its reduced efficiency with a consequent reduced rate of survival free of the disease. Therefore, for the assessment and treatment of these patients to be effective, it is imperative that oncological and psychiatric staff work together.

Note that in addition to considering the patients, their social support network should also be taken into account, so that family members and intimate partners are involved in the process of adapting to the treatment with the help of a qualified, multi-professional team. Nurses, as members of these teams, have a key role in the implementation of psychosocial strategies for individual or groups, directed to the optimization of the treatment of depressive symptoms and the promotion of adherence to chemotherapy treatment.
